# The Impact of Neurogenic Lower Urinary Tract Symptoms and Erectile Dysfunctions on Marital Relationship in Men with Multiple Sclerosis: A Single Cohort Study

**DOI:** 10.3390/jcm11195639

**Published:** 2022-09-24

**Authors:** Riccardo Bientinesi, Simone Coluzzi, Filippo Gavi, Viviana Nociti, Carlo Gandi, Filippo Marino, Stefano Moretto, Massimiliano Mirabella, PierFrancesco Bassi, Emilio Sacco

**Affiliations:** 1 Department of Urology, Fondazione Policlinico Universitario Agostino Gemelli IRCCS, Università Cattolica del Sacro Cuore, 00168 Roma, Italy; 2 Institute of Neurology, Fondazione Policlinico Universitario Agostino Gemelli IRCCS, Università Cattolica del Sacro Cuore, 00168 Roma, Italy

**Keywords:** lower urinary tract symptoms, sexual dysfunction, marital relationship, quality of life

## Abstract

Aims: Multiple sclerosis (MS) is an autoimmune and neurodegenerative disease that is characterized by a great variety symptoms. Most MS patients suffer from neurogenic lower urinary tract symptoms (nLUTS) and erectile dysfunctions (ED). The aim this study is to assess the impact of nLUTS and ED on marital relationships in MS patients. Materials and Methods: MS male patients that arrived for our attention were prospectively enrolled in the study. All of the patients were evaluated on an Expanded Disability Status Scale (EDSS), an IIEF-5 for sexual function, an ICIQ-MLUTS for urinary function, and a Dyadic Adjustment Scale (DAS) for marital relationships. The data were analyzed using descriptive and inferential statistical tests in STATA/MP14. Results: The data of 57 male MS patients were eligible. The mean age was 45 (13.7) years, the mean disease duration was 15.49 (7.86) years, and the mean EDSS score was 3.5 (1.89). In total, 33 (57.89%) MS patients reported urine incontinence, of those, 24 (42.11%) reported UUI. The mean DAS score was 74.40 (34.58). The mean IIEF-5 score was 12.40 (8.05). The mean ICIQ-MLUTS score was 71.94 (41.06). The DAS and ICIQ-MLUTS scores were negatively correlated (r = −0.30, *p* < 0.001). The DAS and IIEF-5 were moderately correlated (r = 0.47, *p* < 0.001). The DAS and EDSS were strongly correlated (r = −0.72, *p* < 0.001). A univariate analysis showed that increasing age (*p* < 0.001), a longer disease duration (*p* = 0.029), a higher EDSS score (*p* < 0.001), and a higher ICIQ-MLUTS score (*p* < 0.001) were all significantly associated with lower DAS scores. Conclusions: This study demonstrated the large negative impact that nLUTS and ED due to MS have on patients’ marital relationships, highlighting the importance of a multidisciplinary approach in MS patients.

## 1. Introduction

Multiple sclerosis (MS) is a chronic autoimmune disease of the central nervous system. The pathogenesis is mainly based on an autoimmune inflammatory process that destroys myelin, the sheath that surrounds the nerve fibers of the brain and spinal cord. It is also well known that MS is not just an inflammatory disease, but it is also a neurodegenerative condition [1]. The etiology of MS is multifactorial and include: genetics, diet, infections, and lifestyle, but the main causes remain mainly uncertain [2,3]. MS is characterized by a high variability of symptoms and disease course. The classic relapse-remitting form of multiple sclerosis (RRMS) is characterized by the onset of recurring clinical symptoms which are followed by a total or partial recovery [1]. Symptoms of multiple sclerosis can occur at any age, but in most cases, they occur between the age of 20 and 40, therefore, it is the leading non-traumatic neurological cause of disability in young and middle-aged people in first world countries [4,5]. The neuronal damage that is due to the loss of myelin can cause numerous neurogenic lower urinary tract symptoms (nLUTS), which can be divided into two categories: bladder-emptying phase symptoms (straining to void, hesitancy, dysuria, diminished stream intensity, incomplete emptying, and intermittency) and bladder-filling phase symptoms (nocturia, urinary urgency, frequency, urge and stress incontinence). A further subdivision of the symptomatology is based on the localization of the neuronal damage on the spinal cord. Suprapontine lesions can cause detrusor overactivity and a reduction of bladder capacity, subpontine-suprasacral lesions can cause detrusor overactivity and detrusor-sphincter dyssynergy, and subsacral lesions can cause hypo/acontractile detrusor [6]. However, the multifactoriality of the disease and the multiple locations of the demyelination of the nervous system prevent the predictability of the symptomatology. The level of disability is scored with the Expanded Disability Status Scale (EDSS), which is a useful tool for evaluating different functional systems to measure neurological disability (pyramidal, cerebellar, brain- stem, sensory, bowel, bladder, visual, and cerebral functions) [7]. All of these clinical manifestations impact negatively on the quality of life and are often associated with depressive symptoms, loss of sleep and productivity and, more generally, they cause health deterioration. About 70% of patients with MS agree that nLUTS affects their quality of life in a medium-to-high degree [8]. In addition to nLUTS, another problem that afflicts patients with MS are sexual dysfunctions, such as a decrease of sexual desire, erectile dysfunction, and alterations in the sensitivity of the genital organs; all of these contribute to influence the life of the couple and of the family and the quality of life. The aim of this study is to evaluate the impact of nLUTS and erectile dysfunction in MS male patients in their marital relationships.

## 2. Materials and Methods

This was an observational research project that was containing a retrospective analysis of the patients’ data and they men who were attending our outpatient’s clinic (Policlinico Universitario Agostino Gemelli IRCSS, Rome, Italy) with a diagnosis of multiple sclerosis according to the present McDonald-Criteria. The patients consented to the use of their clinical data. The data were collected between January 2021 and March 2022. We produced datasets of the patients who were attending our urology outpatient clinic and those who had a diagnosis of clinically stable multiple sclerosis in the 3 months prior the clinical visit were included. Patients with permanent a urethral catheterism, a urinary infection, or additional urological or any other medical conditions altering their urinary (e.g., BPH) or sexual function were excluded. Patients who did not complete all of the questionaries were excluded. All patients that were presenting to the clinic during the study period with a diagnosis of multiple sclerosis and in a relationship were included in the study. The datasets included the clinical history of all of the patients, their baseline characteristics, relationship time, current treatments for urinary and sexual dysfunctions and EDSS scores. During the clinical visit, the patients were asked to complete 3 questionnaires, the Dyadic Adjustment Scale (DAS), the International Consultation on Incontinence Questionnaire Male Lower Urinary Tract Symptoms Module (ICIQ-MLUTS) and the International Index of Erectile Function—5 (IIEF-5). All of the questionnaires have been validated in Italian. The DAS has a range of values that are between 30 and 130, and values <89 were considered as them not having a good marital relationship. The IIEF-5 has a score that ranges from 0 to 25, and a score <21 was considered suggestive for an erectile dysfunction. The ICIQ-MLUTS is a questionnaire that is used for evaluating male lower urinary tract symptoms and their impact on the patients’ quality of life (QoL). It contains 23 symptom questions with their own bother score, and it has a 0–20 range for placing the voiding symptoms on a subscale and a 0–24 scale for placing the incontinence symptoms on a subscale. Descriptive statistics including mean, frequency and percentage were used to summarize the data as appropriate and were presented in the form of texts and tables as appropriate. A Pearson’s correlation was used to test the all-linear relationships between the ICIQ-MLUTS, EDSS, DAS and IIEF-5 scores. The association between the baseline characteristics and clinical variables and the ICIQ-MLUTS, EDSS, DAS and IIEF-5 scores was assessed with a univariate and multivariate linear regression model, with DAS representing the outcome variable. The input variables were age, disease duration, ICIQ-MLUTS, EDSS, IIEF-5, education, and occupation. All of the data were entered and analyzed using STATA/MP 14.0 (StataCorp LLC, 4905 Lakeway Drive, College Station, Texas, USA).

## 3. Results

Data of 57 male MS patients were eligible. Their baseline characteristics are shown in Table 1. The mean age was 45 (13.7) years, the mean disease duration was 15.49 (7.86) years, the mean EDSS score was 3.5 (1.89). Eighty-six percent of all of the MS patients had an EDSS score < 5. The mean DAS score was 74.40 (34.58); 28 (49) MS patients reported that they had marital relationship problems (DAS < 89). The mean IIEF-5 score was 12.40 (8.05). The mean ICIQ-MLUTS score was 71.94 (41.06). In total, 33 (57.89%) MS patients reported urinary incontinence (UI), and of those, 24 (42.11%) reported urgency urinary incontinence (UUI). Storage and voiding symptoms were reported by 36 (63%) and 14 (24%) patients, respectively. Erectile dysfunctions (ED) were reported by 34 (59%) patients. Twenty-eight (49%) and 20 (35%) patients were using pharmacological therapy for LUTS and ED, respectively. The DAS and ICIQ-MLUTS scores were negatively correlated (r = −0.34, *p* < 0.001). The DAS and IIEF-5 were moderately correlated (r = 0.46, *p* < 0.001). The DAS and EDSS were strongly correlated (r = −0.72, *p* < 0.001). The univariate analysis showed that increasing age (*p* < 0.001), a longer disease duration (*p* = 0.029), a higher EDSS score (*p* < 0.001) and a higher ICIQ-MLUTS score (*p* < 0.001) were all significantly associated with lower DAS scores. Being employed or a student (*p* < 0.024) and having higher IIEF-5 scores (*p* < 0.001) were significantly associated with higher DAS scores (Table 2).

## 4. Discussion

MS is a demyelination disease of the central nervous system (CNS) that causes the impairment of the electrical signals through the axonal pathways. This causes different neurological symptoms and urological dysfunctions. In our study, more than half of the MS patients reported to have some form of UI, and of those patients, two-third reported a UUI, thereby concurring with the literature where the prevalence of UUI in MS patients ranges from 37% to 99% [7]. All of the patients reported to have some degree of LUTS. More than half of our patients presented storage symptoms. It has been assessed, and it is in line with our findings, that 80–90% of patients with MS will suffer from some form of LUTS over the course of the disease. These data are of concern because it is known that LUTS are inversely associated with quality of life [9]. Our data showed that LUTS (as measured by ICIQ-MLUTS) and higher disability scores (as measured by EDSS) are negatively correlated with the quality of the marital relationship. The literature reports that LUTS and UI become progressively more severe as the overall neurological disability accumulates, this has an increasing negative impact on the quality of life [10]. Moreover, our results suggest that not only LUTS, but also erectile dysfunction (as measured by IIEF-5) have a strong impact in the quality of the marital relationship. LUTS and UI can limit a patient’s ability to socialize, to be part of intimate relationships or to be able to perform day-to-day household tasks. These could result in marital problems being a plausible explanation of our results suggesting that MS patients that are employed or are students have better relationships. Zonić-Imamović et al. [11] demonstrated that improving the symptoms of UUI caused a significant increasing in the parameter of the quality of life. Therefore, treating LUTS and UI symptoms should be a primary objective in the management of MS patients [12]. However, there are no further specific studies regarding the correlation between LUTS, neurogenic bladder and/or sexual dysfunctions and marital relationship that are measured by a DAS. In this manner, our study represents a pioneering study. Precise and early diagnosis, prescription of medication and implementation of coping strategies are essential tools to contribute to MS patients’ quality of life [13]. It is known that LUTS progressively worsen in MS patients and the management of it is an important challenge for both patients and caregivers. ED is a common but usually overlooked symptoms in MS patients. Eighty percent of our patients reported some level ED, and the majority were open to treatment options. ED should be assessed during the follow-up as they have an important impact on the quality of life and the marital relationship [14]. Education and coping strategies can be explained to the patients and their caregivers in order to improve symptom management [15]. LUTS and ED in MS patients may be assumed to represent a minor problem when they are compared to other neurological deficits, but despite what the literature suggests, such prejudices preclude the possibility to achieve optimal symptom management in these patients. Possible limitations of this study are the patients’ subjective perception of the investigated symptoms and the small number of patients that are in it. However, further studies with a larger number of patients are necessary and are ongoing at our institution in order to produce more detailed data.

## 5. Conclusions

This initial study demonstrated the large negative impact that LUTS and sexual dysfunctions due to multiple sclerosis have on the patients’ marital relationship, as measured by the DAS. Because of the symptoms’ heterogeneity and patient’s expectations, a multidisciplinary approach is required to individually tailor the treatment options, thereby focusing on the patients’ quality of life. A more extensive study on a greater number of patients is certainly necessary to produce even more corroborating data.

## Figures and Tables

**Table 1:** Baseline characteristics.

Variable	Pts ^a^: *n* = 40	Standard Deviation	Lower Limit	Upper Limit
Age (Years)	45 ^b^	13.76	19	70
Disease duration (Years)	15.49 ^b^	7.86	1	30
Gender, no. (%)				
Male	57 (100)	-	-	-
Education, no. (%)				
Middle school	11 (19.30)	-	-	-
High school	30 (52.63)	-	-	-
University	16 (28)	-	-	-
Social status, no. (%)				
Relationship	23 (58)	-	-	-
Married	24 (42)	-	-	-
Occupation, no. (%)				
Unemployed	22 (38.60)	-	-	-
Employed	28 (49)	-	-	-
Student	7 (12.28)	-	-	-
EDSS, mean	3.5	1.89	1	8
Range 1–3; no. (%)	29 (51)	-	-	-
Range 3.5–5; no. (%)	20 (35)	-	-	-
Range 5.5–7; no. (%)	6 (11)	-	-	-
Range 7.5–8; no. (%)	2 (3)	-	-	-
DAS, mean	74.40	34.58	10	120
DAS > 89; no. (%)	29 (51)	-	-	-
DAS < 89; no. (%)	28 (49)	-	-	-
IIEF 5, mean	12.40	8.05	0	25
ICIQ-MLUTS, mean	71.94	41.06	1	168
Incontinence, no. (%)				
Present	33 (57.82)	-	-	-
Absent	24 (42.18)	-	-	-
UUI, n. (%)	24 (42.11)	-	-	-
SUI, n. (%)	2 (3.50)	-	-	-
MUI, n. (%)	7 (12.28)	-	-	-

EDSS: Expanded Disability Status Scale; DAS: Dyadic adjustment scale; IIEF: International Index of Erectile Function; ICIQ-MLUTS: International Consultation on Incontinence Questionnaire Male Lower Urinary Tract Symptoms Module; UUI: urge urinary incontinence; SUI: stress urinary incontinence; MUI: mixed urinary incontinence; ^a^: patients; ^b^: mean.

**Table 2:** Univariate and multivariate linear regression analysis for DAS score.

	DAS Scores					
	Univariate			Multivariate		
Variable	R^2^	B	P	R^2^	B	P
Age	0.112	−0.88	<0.01	0.627	−0.43	<0.39
Education	0.001	2.10	0.757		−1.86	0.72
Occupation	0.089	15.4	0.024		7.27	0.18
Social status	0.001	0.67	0.943		7.98	0.27
Disease duration	0.084	−1.27	0.029		1.13	0.21
EDSS	0.530	−13.2	<0.01		−11.49	<0.01
ICIQ-MLUTS	0.118	−0.29	<0.01		−0.16	0.09
IIEF-5	0.213	1.98	<0.01		0.80	0.08
Incontinence	0.010	−7.0	0.455		9.04	0.25

B, standardized regression coefficient; EDSS, Expanded Disability Status Scale; DAS: Dyadic adjustment scale; IIEF: International Index of Erectile Function; ICIQ-MLUTS: International Consultation on Incontinence Questionnaire Male Lower Urinary Tract Symptoms Module.

## Data Availability

Not applicable.
